# Psychological implication of student unrest on student leaders: A social support perspective

**DOI:** 10.1016/j.heliyon.2023.e22334

**Published:** 2023-11-14

**Authors:** Bunmi Isaiah Omodan

**Affiliations:** Faculty of Education, Butterworth campus, Walter Sisulu University, South Africa

**Keywords:** Student unrest, Psychological implications, Student leaders, Social support

## Abstract

The study explored the psychological implications of student unrest on student leaders in South African universities, focusing on both the negative and positive implications. The study is underpinned by social support theory and lensed within transformative paradigm. The study is qualitative in nature and employs participatory research design. Three universities were randomly selected across three provinces in South Africa, and four student leaders from each univesity were selected as participants using convenient sampling technique. Semi-structured interview was used to elicit information from the participants, and the data was analysed using thematic analysis. The study found that social and unpleasant anxiety and social group conflict tendencies are major negative psychological implications on student unrest. On the other hand, it also revealed that student unrest enhances the sense of empowerment and solidarity and also promotes self-esteem and political awareness among student leaders, with a recommendation that universities should provide adequate social support for student leaders.

## 1 Introduction

Student unrest is a common occurrence in universities around the world, and South African universities are no exception. In this study, both student unrest and student protest are used synonymously. However, there have been notable student unrest in the history of South Africa. Among these is #FeesMustFall (2015–2017) - The protests began at the University of the Witwatersrand in Johannesburg and spread to other universities across South Africa, and the primary reason for the protests was to demand a halt to the rising cost of higher education in the country [1,2]. #RhodesMustFall also another protest where students demanded the removal of the statue of Cecil John Rhodes, a British colonialist and mining magnate, from a university campus [3,4]. Another notable protest is the popular #Outsourcing Must Fall that took place at various universities across South Africa to demand an end to the outsourcing of campus support staff, such as cleaners and security guards, who were often paid low wages and lacked job security [5].

Apart from these national student movements, virtually all universities have faced different degrees of unrest related to these issues in some form or another. Examples include the University of Zululand, the University of Fort Hare, the University of Pretoria, North-West University, Tshwane University of Technology, and the University of Cape Town [6,7]. These forms of unrest can often be traced back to deficiencies in communication among the university leaders and students, hike in tuition fees, and hostel accommodation, among others [[8], [9], [10], [11]].

However, the causes of student unrest are not the focus of this study; rather, the study focuses on the implications of student unrest and/or protests on the student leaders. This is essential because student leaders are often at the forefront of student unrest, and as such, they bear the brunt of the psychological effects of such unrest. Student leaders in this study are who hold formal leadership positions within the university and in most cases referred to as Student Representative Council (SRC). And they are the ones who are expected to negotiate with university authorities, organise protests, and communicate with their fellow students. As a result, they are more likely to experience high levels of stress, anxiety, and depression. To support this argument, studies confirmed that students in South African universities experienced stress and anxiety, which were attributed to the pressure of their responsibilities, academic demands, and financial difficulties [12,13]. Also, being at the forefront of the protest, student leaders are likely to experience feelings of isolation and stigmatisation. This is because they are often seen as troublemakers and rebels by university authorities and some members of the public, which can lead to feelings of loneliness and isolation, as well as social and emotional withdrawal [14]. In the same vein, a study by Ref. [15] confirms that student unrest negatively affects student mental health.

Moreover, student leaders may also experience trauma and emotional distress as a result of their involvement in student unrest. This is because they may be subjected to physical and emotional violence, intimidation, and harassment from university authorities, police, and other parties. For example, during the #FeesMustFall protests in 2015 and 2016, many student leaders were arrested, assaulted, and subjected to tear gas and rubber bullets by police [16]. In addition, student leaders are also vulnerable to academic imbalance because they may miss classes, assignments, and exams due to their involvement in protests and demonstrations.

The above literature justifies that South African university student leaders deal with unfathomably difficult and pressuring conditions during protest activities. With the backdrop of existing workloads, high fees, and contractual obligations, to name a few, student leaders are at risk of experiencing a range of psychological implications. These may range from stress and anxiety to depression, feelings of isolation and stigmatisation, trauma and emotional distress. Making the precarious situation more difficult is the fact that such effects often go unnoticed by researchers and university management. Therefore, further study is needed to identify ways in which to support current student leaders or even mitigate similar distress experienced by future leadership. The argument here is that the psychological implications of student unrest on student leaders in South African universities are significant and often overlooked. Hence, this study provides knowledge on the psychological effects of student unrest on student leaders in South African universities.

### 1.1 Theoretical Framework

Social support theory was adopted to underpin this study. Social support theory is a psychological theory that explains how social networks and interpersonal relationships can have a positive impact on a person's mental and physical well-being [17,18]. The theory emerged from a combination of research on interpersonal relationships, theories of well-being and empirical studies of health outcomes. Since then, researchers have argued numerous ways in which social support is associated with better physical and mental health outcomes as well as success in work and school [19,20]. For example, individuals with higher levels of supportive relationships tend to be more productive in their tasks, have an improved likelihood of being promoted at work, cope better when faced with challenges or loss, experience greater self-acceptance and satisfaction in life, and have lower levels of anxiety or depression.

The theory assumes that social support is a form of social exchange [21]. That is, individuals provide support to others in their social network in exchange for support when they need it. According to this theory, such support includes emotional support (e.g., listening and providing empathy), informational support (e.g., providing advice or information), and tangible support (e.g., providing practical assistance) [22,23]. The theory also assumes that social support can, directly and indirectly, affect an individual's health and well-being [24,25]. Direct effects refer to the immediate impact of social support on an individual's health or well-being, while indirect effects refer to the ways in which social support can buffer the effects of stressors and protect individuals from negative health outcomes. However, this theory opines that social support can come from a variety of sources, including family members, friends, colleagues, and even pets [26]. The quality and quantity of social support that an individual receives can vary, and individuals may need different types of social support depending on their needs and circumstances. Based on the above, one can conclude that social support theory suggests that social support is an important resource that can help individuals cope with stress, promote physical and mental health, and improve overall well-being.

Since social support theory posits that people require both tangible and emotional help from their social network to cope with stress, this theory appears to be particularly relevant as people are becoming more reliant on social and network relationships. As student leaders become increasingly isolated, they rely on these interpersonal connections to facilitate stress relief as well as emotional sustenance to recover from psychological implications arising from student unrest. As humans are prone to vulnerability after sustained periods of social deprivation, social support theory helps explain how communication and closeness within any group can impart a sense of emotional well-being compared to being deprived of interpersonal interaction entirely. By explaining the positive impacts of social engagement on mental orientation and comfort, this theory is invaluable for understanding human behaviour during difficult times, most especially the student leaders during and after the protest.

### 1.2 General research question

Based on the above problems, the stud answers the following research question.

• What is the psychological implication of student unrest on student leaders in South African universities?

### 1.3 Research objectives

To answer the above general questions, the following research objectives were raised to guide the study. That is, the study.

• Investigates negative implications of student unrest on student leaders in South African universities.
• Explores the positive implication of student unrest on student leaders in South African universities.

## 2 Methodology

This section discusses the research paradigm and approach and design alongside other methods, including ethical considerations.

### 2.1 Research paradigm

This study is lensed by transformative paradigm. It is a type of worldview that seeks to shift traditional power structures in order to benefit marginalised individuals and communities [27]. It emphasises a holistic approach to transformative change, which includes equitable redistribution and empowerment of traditionally underrepresented or disempowered groups and provision of access to basic needs [28]. Another important aspect of the transformative paradigm is the focus on social justice and the empowerment of marginalised groups [29]. This involves examining the power dynamics that exist in society and working to address systemic inequalities and give voice to those who are typically silenced to challenge the status quo. Most importantly, the paradigm recognises that the co-researcher is not an objective observer but an active participant in the research process [30]. That is, the goal of the transformative paradigm is not just to understand the phenomenon being studied but also to bring about positive change in the lives of the research participants. Hence the need for a participatory research design that will enable the participants to come together and find solutions to the problem.

### 2.2 Research approach

The study adopts a qualitative research approach. Qualitative research is renowned due to its ability to provide a more detailed and in-depth analysis of a problem or particular issue [31,32]. The advantages of qualitative research include an opportunity for researchers to investigate topics from different angles, delve deep into the subject matter of their study, develop theories, and analyse data from everyday experiences, which have become increasingly popular within various disciplines [33]. Given these benefits and its ability to uncover meanings perceived by participants on their own terms, this current study chooses to adopt a qualitative approach. It is also relevant because it focuses on understanding the attitude, behaviour, value and meaning that people attach to their actions or behaviour, enabling researchers to put together a deeper understanding of a particular topic. In this case, it enables the participants to provide uncompromised information about the implication of student protest on their persons. As such, it facilitates an in-depth narrative for gaining greater insight and detail about our topic of interest.

### 2.3 Research design

Participatory research (PR) is adopted as a research design. This design involves inviting participants who hold positions of expertise and local knowledge to meaningfully engage in a dialogue with researchers [34,35]. That is, PR allows both parties to co-create the research design, making it more authentically reflective of the subject being studied. Engaging participants in this manner ensures that their unique expertise can be applied to outcomes with greater precision and accuracy than what would otherwise be possible by relying on only traditional models of research [36]. Ultimately, PR offers possibilities for an in-depth exploration of complicated topics, providing insights and solutions which cannot be unlocked without its application.

PR was used to engage student leaders as active participants in addressing the psychological impacts of student unrest on their well-being. Through PR, student leaders collaborated with the researchers to co-create effective solutions that considered their unique perspectives and experiences. By involving student leaders in this process, the study fosters a greater sense of agency and empowerment [37] among those most affected by student unrest. Overall, the use of PR in this study highlights the value of including diverse voices and perspectives in research to create more meaningful and effective interventions.

### 2.4 Participants and selection of participants

Drawing from the collective experiences of student leaders, three universities were randomly selected across three provinces, and participants were evenly distributed among them. Twelve participants were chosen from the Student Representative Council (SRC) member due to their familiarity with experiences related to the topic of study and active involvement in the protest. That is, student leaders were integral to the study due to their role in instigating student protests and their firsthand experience with the issues under investigation. Convenient sampling was utilised to select twelve student leaders for the study. This approach was deemed appropriate as it allowed for the inclusion of any available student leaders [38] and actively involved in the issue of student unrest, providing a diverse range of perspectives. Their year of experience in student activism was also considered. Among the twelve selected student leaders, four were chosen from each of the three participating universities to ensure that multiple viewpoints were represented. Through interviews with these student leaders, the study gains a deeper understanding of the psychological implications of student unrest on those most directly involved in leading and participating in these protests.

Table 1 illustrates the distribution of participants across three universities (A, B, C), each having four student leaders. Their active experiences vary from 2 to 4 years. University A has student leaders with 2, 3, 4, and 3 years of active experience, respectively. University B's student leaders each have 3, 4, 4, and 3 years of active experience, respectively. Lastly, the student leaders from University C have 4, 3, 2, and 3 years of active experience, respectively.

**Table 1:** Participants' distribution table.

University	Participants	Active Experience
A	4 Student leaders	P1: 2 years
		P2: 3 years
		P3: 4 years
		P4: 3 years
B	4 Student leaders	P1: 3 years
		P2: 4 years
		P3: 4 years
		P4: 3 years
C	4 Student leaders	P1: 4 years
		P2: 3 years
		P3: 2 years
		P4: 3 years

### 2.5 Method of data collection

The data collection instrument employed in this study was a semi-structured interview, which was chosen because of its effectiveness in qualitative and participatory research approaches. This method allowed researchers to elicit valuable insights and perspectives from study participants, enabling them to share their experiences and thoughts on the issues being investigated. By following a structured approach, researchers were able to ask predetermined questions that focused on understanding the problems and potential solutions. This is consistent with the findings of [39], who note that semi-structured interviews are useful for gaining a coherent and comprehensive understanding of complex issues. However, the use of semi-structured interviews allowed for a detailed exploration of the research questions and a deeper understanding of the experiences and viewpoints of the participants on both the positive and negative implications of student unrest on them. To ensure the appropriateness of the interview questions, the researcher tested the interview questions with a small group of dummy participants to ensure they were clear and comprehensible before the actual interview with the selected participants.

### 2.6 Method of data analysis

The study utilised thematic analysis to analyse the qualitative data, which involves identifying and understanding recurring themes within the data. Thematic analysis is commonly used to analyse data from interviews, observations, and focus group discussions. By identifying and coding common themes and patterns that emerge from the data, researchers can gain insights into the experiences of participants and understand how the themes relate to one another. The six steps of thematic analysis outlined by Refs. [40,41] included becoming familiar with the data, identifying initial codes, searching for or identifying themes, reviewing the themes, defining and naming the themes, and writing the report used. This method of analysis was followed, and it allowed the researcher to analyse the data based on the objectives that were already divided into themes and gain a deeper understanding of the overall meaning of the data. This is, therefore, a valuable method because it enables me to analyse qualitative data obtained from student leaders to produce an informed meaning of the participants' experiences.

### 2.7 Ethical considerations and member checking

The research ethics pertaining to this issue were duly adhered to and upheld. The study was granted ethical clearance by Walter Sisulu University's ethics committee under protocol number FEDREC 03-11-21. Prior to their involvement, the participants were given a comprehensive explanation of the research, its procedures, and the approaches employed, and their consent was sought. They expressed their willingness to participate voluntarily and were given the option to withdraw at any time should they feel uneasy. The participants' identities were verified and protected, with their responses being assigned coded pseudonyms (P1 to P12) to ensure anonymity.

To ensure the accuracy of the identified themes, member checking was implemented with a rigorous process involving the active involvement of participants or stakeholders. I allow the participants to review and validate the findings, interpretations, and conclusions derived from the data. I also encouraged open communication by seeking their feedback and input to ensure the analysis's accuracy, completeness, and authenticity. And lastly, I prioritised their perspectives by ensuring that their voices were incorporated into the final research outputs to enhance the validity and credibility of the findings.

### 2.8 Data presentation and analysis

The data analysis presented in this section follows the principles of thematic analysis outlined earlier, and the data is organised according to the research objectives. Table 2 displays the two themes for each objective, which are derived from the participants' statements.

**Table 2:** Thematic representation of data.

General Question	Objectives	Sub-Themes
What is the psychological implication of student unrest on student leaders in South African universities?	Investigates negative implications of student unrest on student leaders in South African universities.	• Social and unpleasant anxiety
		• Social group conflict tendencies
	Explores the positive implication of student unrest on student leaders in in South African universities.	• Sense of empowerment and solidarity
		• Promote self-esteem and political awareness

#### 2.8.1 **Objective 1, theme 1:** social and unpleasant anxiety

Based on the data collected from the participants, it is obvious that there are many negative implications of student unrest on the well-being of the student leaders who mostly lead student protests in the universities. That is, there is evidence to suggest that participants are experiencing social and unpleasant anxiety during and after the student protests. This is shown in the below statements.

P1: "I worry about the potential consequences of participating in protests. There's always a risk of getting arrested or facing backlash from those in power, and it's important to weigh the risks and benefits before getting involved."

P2: "I find it difficult to concentrate on my studies during the protests. My mind is so preoccupied with everything that's going on that it can be hard to focus on anything else, even the anxiety is sucking."

P3: "The fear and anxiety I feel when faced with the police presence during protests can be really overwhelming. It's like my fight or flight response is constantly activated, which can be exhausting both during and after the protest."

P4: "The emotional toll of participating in protests can be really challenging. I sometimes struggle with feelings of anger, frustration, and sadness, which can be draining both during and after the demonstrations."

From the statement above, P1 expresses worry about the potential consequences of participating in protests and the need to weigh the risks and benefits before getting involved. This suggests that the individual is aware of the potential risks and is experiencing anxiety related to participating in protests. In the same vein, P2 mentioned the difficulties in concentrating on studies during protests and experiencing preoccupation with everything that's going on, which can be hard to focus on anything else. This indicates that the individual's attention and focus are impacted, potentially due to anxiety and stress related to the protests. The statement of P3, which describes the feeling of overwhelming fear and anxiety when faced with police presence during protests, also suggests that the individual is experiencing intense and persistent anxiety related to the protests. The statement of P4 is not also far from others, with an argument that individuals experience a range of negative emotions related to the protests, which can be emotionally taxing. The following statements also support the presence of various forms of anxiety.

P7: "I sometimes feel like I'm taking a risk by participating in protests, and that can be really stressful. It's like I'm putting myself in harm's way to fight for something I believe in."

P8: "Navigating the political dynamics of protests can be really stressful. It can be hard to know who to trust or what alliances to form, and that can be mentally and emotionally exhausting."

P1: "After the protest, I sometimes feel like I need to take a break from everything. It's like my mind and body are exhausted from all the emotional and mental energy that went into the demonstrations."

The three statements above suggest that individuals who participate in protests experience various levels of stress, anxiety, and exhaustion. Evidence from P7 implies that participating in protests involves taking risks that can be stressful and anxiety-inducing. While P8 also lament that navigating the political dynamics of protests can be mentally and emotionally exhausting. On the of P1, protesting takes a toll on one's energy and well-being.

Based on this, it is possible to deduce that student leaders face social and unpleasant anxiety during and after protests. The anxiety is related to a range of factors, including the potential risks and consequences of participating in protests, the impact of the protests on attention and focus, fear and anxiety related to police presence, emotional toll and negative emotions related to participating in protests, stress related to navigating the political dynamics of protests, and exhaustion related to the emotional and mental energy required for participation. This finding is consistent with previous research that organisational conflict leads to anxiety among employees [42,43], suggesting that social unrest, such as protests or other forms of organisational conflict, can cause anxiety in participants. Omodan argued that when individuals perceive a threat to their well-being, it can lead to feelings of anxiety and stress [44,45]. Similarly [46], discussed the psychological implications of organisational conflict on social well-being, arguing that the stress and anxiety caused by conflict can have negative impacts on individuals' mental health and social well-being.

#### 2.8.2 **Objective 1, theme 2:** social group conflict tendencies

Based on the data collected from the participants, one of the negative implications of student unrest appears to be the possibility of group conflict between the student leaders themselves. That is, there is evidence to suggest that participants disagree and demonstrate differences during student protests.

P9: "The negative reactions and backlash from those who don't support our cause can be really hurtful. It can feel like our efforts are being dismissed or belittled, which can be emotionally challenging both during and after the protest”.

P2: "I've found that there can be a lot of tension and conflict within the student body during protests. Everyone has their own ideas and opinions, and sometimes it can be difficult to find common ground and work together."

P3: "Sometimes it's hard to keep up with all the information and updates about the protest movement. There are so many different groups and events going on, and it can be overwhelming to try and stay in the loop."

The statement from P9's indicates that there is always negative reactions and backlash from those who oppose their cause. It suggests that such reactions can feel dismissive and belittling, making it challenging to sustain motivation and engagement in the protest movement. In the same view, P2's statement points out the potential for tension and conflict within a protest movement, particularly when there are diverse opinions and ideas. And P3's statement emphasises the challenge of keeping up with the fast-paced and constantly evolving nature of protest movements. It underscores the need for effective communication and coordination to ensure that everyone is informed and able to participate in meaningful ways. Not so far from these are statements below.

P4: "I sometimes feel isolated and unsupported when I participate in protests. It can be hard to find others who share my beliefs and are willing to stand up for what's right."

P5: "It can be difficult to navigate the political landscape during protests. There are often power dynamics at play and different interest groups vying for influence, and it's hard to know who to trust or align with."

P6: "One of the challenges I've encountered is dealing with negative reactions from those who don't support our cause. It can be discouraging to hear people dismiss our efforts or belittle our beliefs."

The above statements relate to the challenges of participating in protests and activism. The first statement by P4 expresses the difficulty of finding a supportive community that shares one's beliefs and values, which can result in feelings of isolation and lack of support. The second statement (P5) acknowledges the complex political dynamics that can arise during protests, with different interest groups vying for influence and making it difficult to know who to trust. The statement of P6 also supports highlights the emotional impact of encountering negative reactions from those who do not support one's cause, which can be discouraging and disheartening.

The finding here is that there is social group conflict is one of the negative psychological implications of student unrest on student leaders. This justifies that there is various social interest even among or within the student during the protest, which constitutes an unpleasant situation for student leaders to face. The implication is that students who engage in activism and protests may struggle with finding a supportive community, navigating the complex political landscape, and coping with negative reactions from those who do not support their cause. These challenges are discouraging and may lead to feelings of isolation and lack of support [47]. This finding also justifies the need to address the issue of power dynamics and find ways to build a more inclusive and collaborative management system that can bring together diverse groups of people who share common goals and values [48].

#### 2.8.3 **Objective 2,** theme **1:** sense of empowerment and solidarity

In order to respond to objective 2, the data collected from the participants shows some positive implications of student unrest on student leaders. Among these implications is that the student unrest enables the student leaders to be empowered and feel some sense of solidarity. The is evidenced in the below participants’ statements.

P1: "During the protest, I feel a sense of empowerment, and there is a sense of solidarity with my fellow students. It's like we're all working together to create positive change."

P3: "Being part of a protest also makes me feel like I'm part of something bigger than myself. It's not just about my own beliefs, but about standing up for what's right for our community and society as a whole."

P4: "Protests are also a way for me to use my voice and have it be heard. It's an opportunity to make a statement and show that there are people who care about these issues and demand change."

P11: "Despite the fear, though, participating in protests can also be empowering. It's like I'm facing my fears head-on and proving to myself that I have the courage to stand up for what's right."

These participants share similar views on the empowering and positive effects of participating in protests. They describe feeling a sense of solidarity with fellow protesters and being part of something bigger than themselves, such as a community or society. Furthermore, protests provide an opportunity for individuals to use their voices and make a statement about the issues that matter to them. P11's statement about facing fears also highlights the courage required to take part in protests and suggests that overcoming these fears can be empowering. Overall, the participants view protests as a way to effect positive change, both for themselves and for the wider community. In the same vein, the following participants also share the same sentiments.

P5: "Participating in a protest is a chance to learn more about the issue at hand, to listen to different perspectives, and to engage in meaningful discussions with others who are passionate about creating change."

P6: "during protests, I have some sense of courage to raise awareness and start a conversation about issues that are often overlooked, even if they are not the same as the issues we are dealing with."

P12: "For me, protests make to feel the pain o the past comrades and honour the legacy of those who have fought for justice and equality before us. Yhoo, it is sometimes interesting."

The participants above are on the same views, starting from P5, who confirmed that participating in protests offers a valuable learning experience, allowing individuals to gain a deeper understanding of the issue, hear different perspectives and engage in meaningful discussions with like-minded individuals. P6 also highlights the empowering nature of protests, even if the issue at hand is not directly related to the cause, as it provides an opportunity to raise awareness, start conversations and spark change. Statement from P12 reveals that protesting honours the legacy of those who fought for justice in the past and creates a sense of connection to the cause. The last sentence of P12, "Yhoo, it is sometimes interesting," suggests that the speaker finds the experience of protesting to be intellectually stimulating and/or emotionally intense.

Based on this analysis, the study found that student leaders feel some sense of empowerment and solidarity during and after student protests. This is because they see it as an opportunity to create positive change and stand up for what is right, even in the face of fear. They also feel like they are part of a larger community that is working towards a common goal of advocating for their beliefs and promoting societal change. Additionally, protests are seen as a way for students to use their voices and to be heard on important issues. This is consistent with the argument that student activism is powerful to contribute to the greater good and effect change in the student community and societal change [49,50].

#### 2.8.4 **Objective 2, theme 2:** promote self-esteem and political awareness

The analysis of the collected data showed that the participants agreed that student unrest could positively impact their self-esteem and political awareness. That is, engaging in protests and demonstrations has personal and social benefits for student leaders. Evidence is shown below.

P9: "Despite the emotional challenges, though, participating in protests can also be incredibly fulfilling. It's like I'm doing something that's truly meaningful and that aligns with my values, which can be incredibly satisfying."

P10: "Even with this kind of fear, participating in protests can also be a source of courage and bravery. It's like I'm proving to myself that I have the strength to stand up for what's right, even in the face of adversity."

P11: "During protests, I feel like I'm part of something bigger than myself. It's like I'm part of a movement that's working towards positive change, and that can be incredibly motivating."

P7: "Despite the self-doubt, though, participating in protests can also be a source of pride and self-esteem. It's like I'm living up to my own values and principles, which can be incredibly affirming."

P12: "Despite the challenges, though, participating in protests can also be a source of learning and growth. It's like I'm developing my own political awareness and skills, which can be incredibly valuable for my future endeavours."

These statements highlight the personal and emotional benefits of participating in protests. From the above data, P9 suggests that while protests can be emotionally challenging, they can also be fulfilling, providing a sense of meaning and alignment with personal values. In the same view, P10 adds that protests can be a source of courage and bravery, allowing individuals to prove to themselves that they have the strength to stand up for what is right. P11 also notes that protests can create a sense of connection to a larger movement, providing motivation for positive change. The statement P7 support other participants that protests can be a source of pride and self-esteem, allowing individuals to live up to their values and principles. P12, on the other hand, also reiterated the positive impact of protesting on personal growth and development, including the acquisition of political awareness and skills that can be valuable for future endeavours.

The above analysis revealed that student unrest promotes self-esteem and political awareness among student leaders. These statements suggest that while protesting can be emotionally challenging, it also provides an opportunity for personal growth and development. Its psychological benefits include a sense of purpose, courage, motivation, and pride. Several studies have shown that engaging in political activism, including protesting and other forms of social movement participation, can have positive psychological effects on individuals, such as increased self-esteem and a sense of purpose [[51], [52], [53]]. Additionally, such student activism can enhance political awareness and increase engagement with political and social issues [54].

## 3 Conclusion and recommendations

The study investigated the psychological effects of student unrest on student leaders in South African universities. The study examined both the negative and positive implications of student unrest on student leaders. From the findings of the study, it was concluded that social and unpleasant anxiety and social group conflict tendencies were the major negative psychological implications of student unrest. However, the study also concludes that student unrest promotes a sense of empowerment, solidarity, self-esteem, and political awareness among student leaders. To address the potential negative impact of student unrest on student leaders, the study recommended that universities in South Africa provide adequate social support for student leaders. The study emphasised that the provision of social support for student leaders is essential and can help them cope with both physical and mental stress and improve their overall well-being. The social support system includes emotional support, such as listening and providing empathy, informational support by providing advice or information; and tangible support, by providing practical assistance. By providing social support, universities can help student leaders navigate the emotional challenges associated with participating in student unrest and enhance their psychological well-being. This is in line with the postulations of social support theory which agitated that social support systems which include emotional support (e.g., listening and providing empathy), informational support (e.g., providing advice or information), and tangible support (e.g., providing practical assistance) is required and can help individuals cope with both physical and mental stress, and improve overall well-being.

### 3.1 Limitation

It is crucial to acknowledge that the scope of this study was confined to student leaders within South African universities, thereby limiting the generalizability of the findings to other contexts. Moreover, the study's reliance on a relatively small sample size introduces the possibility of biased outcomes, thus necessitating recognition of this limitation within the present research.

## Data availability statement

The author does not have permission to share data.

## CRediT authorship contribution statement

**Bunmi Isaiah Omodan:** Writing – review & editing, Writing – original draft, Resources, Project administration, Methodology, Investigation, Funding acquisition, Formal analysis, Data curation, Conceptualization.

## Declaration of competing interest

The authors declare that they have no known competing financial interests or personal relationships that could have appeared to influence the work reported in this paper.
